# DNA methylation landscape of hepatoblastomas reveals arrest at early stages of liver differentiation and cancer-related alterations

**DOI:** 10.18632/oncotarget.14208

**Published:** 2016-12-25

**Authors:** Mariana Maschietto, Tatiane Cristina Rodrigues, André Yoshiaki Kashiwabara, Érica Sara Souza de Araujo, Talita Ferreira Marques Aguiar, Cecilia Maria Lima da Costa, Isabela Werneck da Cunha, Luciana dos Reis Vasques, Monica Cypriano, Helena Brentani, Silvia Regina Caminada de Toledo, Peter Lees Pearson, Dirce Maria Carraro, Carla Rosenberg, Ana C.V. Krepischi

**Affiliations:** ^1^ Brazilian Biosciences National Laboratory (LNBio), Brazilian Center for Research in Energy and Materials (CNPEM), Campinas, Brazil; ^2^ Department of Genetics and Evolutionary Biology, Institute of Biosciences, University of São Paulo, São Paulo, Brazil; ^3^ Universidade Tecnológica Federal do Paraná, Campus Cornélio Procópio, Paraná, Brazil; ^4^ International Research Center, A. C. Camargo Cancer Center, São Paulo, Brazil; ^5^ Department of Pediatric Oncology, A. C. Camargo Cancer Center, São Paulo, Brazil; ^6^ Department of Pathology, A. C. Camargo Cancer Center, São Paulo, Brazil; ^7^ Department of Pediatrics, Pediatric Oncology Institute (GRAACC), Federal University of São Paulo, São Paulo, Brazil; ^8^ Department of Psychiatry, School of Medicine, University of São Paulo, São Paulo, Brazil

**Keywords:** DNA methylation, embryonal tumor, hypomethylation, cell differentiation arrest, hepatoblastoma

## Abstract

Hepatoblastomas are uncommon embryonal liver tumors accounting for approximately 80% of childhood hepatic cancer. We hypothesized that epigenetic changes, including DNA methylation, could be relevant to hepatoblastoma onset. The methylomes of eight matched hepatoblastomas and non-tumoral liver tissues were characterized, and data were validated in an independent group (11 hepatoblastomas). In comparison to differentiated livers, hepatoblastomas exhibited a widespread and non-stochastic pattern of global low-level hypomethylation. The analysis revealed 1,359 differentially methylated CpG sites (DMSs) between hepatoblastomas and control livers, which are associated with 765 genes. Hypomethylation was detected in hepatoblastomas for ~58% of the DMSs with enrichment at intergenic sites, and most of the hypermethylated CpGs were located in CpG islands. Functional analyses revealed enrichment in signaling pathways involved in metabolism, negative regulation of cell differentiation, liver development, cancer, and Wnt signaling pathway. Strikingly, an important overlap was observed between the 1,359 DMSs and the CpG sites reported to exhibit methylation changes through liver development (p<0.0001), with similar patterns of methylation in both hepatoblastomas and fetal livers compared to adult livers. Overall, our results suggest an arrest at early stages of liver cell differentiation, in line with the hypothesis that hepatoblastoma ontogeny involves the disruption of liver development. This genome-wide methylation dysfunction, taken together with a relatively small number of driver genetic mutations reported for both adult and pediatric liver cancers, shed light on the relevance of epigenetic mechanisms for hepatic tumorigenesis.

## INTRODUCTION

DNA methylation in mammals plays a key role in embryonic and differentiation processes through the modulation of chromatin compaction states leading to domains of variation in transcriptional activities [1]. The Developmental Origins of Health and Disease (DOHaD) hypothesis postulates that environmental factors acting in *utero*, together with genetic factors, may greatly influence disease susceptibility and outcomes in both infancy and adulthood [2, 3], particularly modulating alterations in DNA methylation in pediatric pathological conditions [4].

A specific class of pediatric cancers – the embryonal tumors – is defined based on embryonic features of the tumors and their very early age of onset. A relatively small number of genetic mutations have been reported in pediatric tumors compared to adult solid tumors [5, 6], and this paucity of mutations may be partially correlated with the early age of onset [5]. Another possible explanation is that mechanisms other than DNA mutation may disrupt cell differentiation processes during development, a model that has been proposed for embryonal tumors [7]. Hepatoblastomas are embryonal tumors of the liver and are the most common hepatic tumors of early childhood, with an incidence in the United States estimated to be between 5.2 and 10.5 cases per million children <1 and 1-4 years old, respectively [8]. In Brazil, an age-adjusted incidence rate of 2.8 per million children less than 15 years of age has been reported [9]. Due to their rarity, knowledge regarding genetic predisposing factors for hepatoblastoma is limited. Indeed, few genetic alterations have been found in both cytogenetic and point mutation studies of hepatoblastomas. The identified genetic alterations are primarily linked to the Wnt signaling pathway, with a relatively high frequency of *CTNNB1* activating mutations [10, 11]; its role was demonstrated after insertion of *CTNNB1* mutations in precursor liver cells, resulting in high incidence of hepatocellular carcinomas and hepatoblastomas in mice [12].

A less studied pathway that could partially explain both developmental disruption and cancer development is the occurrence of epigenetic changes [3, 4]. DNA methylation is a stable modification involved in many biological processes, including tissue differentiation, embryogenesis, and disease development [13]. In general, whereas normal cells usually have unmethylated gene promoters and CpG islands, and heavily methylated repetitive sequences, the opposite pattern is found in tumor cells. Anomalous DNA methylation represents an important mechanism driving tumor development and progression [14]. Although the roles of DNA methylation in oncogenesis have not yet been fully elucidated, promoter hypermethylation has been shown to be associated with inappropriate transcriptional repression of tumor suppressor genes, and hypomethylation of repetitive sequences may result in activation of retro-transposon elements and genomic instability [15, 16].

To date, most of studies investigating DNA methylation in hepatoblastomas have focused on specific genes or genomic regions. Site-specific hypermethylation of selected imprinted regions were reported in hepatoblastomas in comparison to normal liver counterparts, suggesting that aberrant DNA methylation is associated to hepatoblastoma formation [17]. Hypermethylation of the promoter regions of *RASSF1A* [18–20], *CASP8* [18], *SOCS1* [21–23], *APC, CDH1, MT1G* [23], *HHIP* [10], *CDKN2A* [24], and *IGF2* [18, 23] were also reported. Additionally, hypomethylation of *IGFBP3* [25] has been described in a subgroup of tumors. To date, only two genome-wide methylation studies in hepatoblastoma samples have been published. In one of them, the comparison of three matched tumor-normal samples revealed genes exhibiting aberrant methylation that were involved with cell adhesion, blood coagulation and nervous system development, in addition to low methylation level near the transcriptional start site of the alpha fetoprotein gene [26]. The second one reported the investigation of hepatoblastomas from two patients, and revealed four genes, *GPR180*, *MST1R*, *OCIAD2* and *PARP6*, with methylation status that could be associated with clinical parameters, such as age at diagnosis and poor outcome [27].

In the present study, we carried out an extensive methylation analysis across the entire genome using two independent cohorts of hepatoblastomas (8 tumors paired with their adjacent normal liver tissues from the same patients, and 11 hepatoblastomas and 3 normal liver tissues from different patients). We examined the DNA methylation status of >450,000 CpG sites, as well as LINE-1 sequences throughout the entire genome. The methylation levels of hepatoblastomas were compared on a *locus* by *locus* basis with those found in non-tumoral liver samples. Furthermore, we interrogated the extent of the overlap between the differentially methylated sites (DMSs) here detected in hepatoblastomas and the CpG sites reported to exhibit methylation changes through liver development.

## RESULTS

### A trend toward global DNA hypomethylation was detected in hepatoblastomas

We analyzed 19 hepatoblastomas using a discovery group (Hepatoblastoma set #1) and a validation group (Hepatoblastoma set #2). This strategy was used to lessen the number of false negatives because we studied a small sample size. The clinical features of the patients with hepatoblastomas are summarized in Table 1.

**Table 1:** Clinical characterization of the 19 patients with hepatoblastomas

ID	Age at diagnosis	Gender	Histology^*^	Treatment Protocol	Recurrence	Metastasis	Overall survival
**HB15**	2.5 years	F	Embryonal	PRETEXT	-	-	1 year
**HB16^#^**	10 months	M	Fetal	PRETEXT III	-	-	> 11 years
**HB17**	3.0 years	F	Fetal	PRETEXT III	-	-	> 10 years
**HB18^#^**	9 months	M	Embryonal	PRETEXT III	-	-	> 9 years
HB28	20.0 years	M	Embryonal	PRETEXT IV	yes	-	1 year
**HB30**	5.5 years	M	Mixed fetal (85%) /embryonal (10%) + small cells (5%)	PRETEXT IV	N/A	yes (lung)	N/A
**HB31**	2.0 years	M	Fetal	PRETEXT	-	-	> 5 years
**HB32**	5 months	F	Embryonal	PRETEXT IV	-	yes (lung)	> 5 years
**HB33^#^**	3 months	F	Fetal	PRETEXT III	-	-	> 2 years
HM35^#^	2.2 years	M	Fetal	PRETEXT III	-	-	> 10 years
HB37	1.1 year	F	Mixed fetal (5%)/embryonal (40%) + tumoral stroma (55%) + non-tumoral stroma (10%)	PRETEXT	-	-	> 7 years
HB38	12.0 years	F	Fetal + non-tumoral stroma (10%)	PRETEXT IV	-	-	> 2 years
HB39	7.0 years	M	Fetal + non-tumoral stroma (30%)	PRETEXT II	-	-	8 months
HB40	2.0 years	M	Fetal	PRETEXT	-	-	> 4 years
HB41	2.0 years	M	Mixed fetal/embryonal + tumoral stroma (55%)	COG	-	yes (lung)	> 2.5 years
HB42	13.0 years	M	Fetal + tumoral stroma (50%)	PRETEXT	-	-	N/A
HB43	1.5 years	M	Embryonal + tumoral stroma (50%)	PRETEXT III	-	-	> 6 years
HB44	4.5 years	M	Embryonal + tumoral stroma (30%)	PRETEXT	-	-	N/A
HB45	5 months	F	Fetal (90%)	PRETEXT	-	-	8 months

Eight cases with matched hepatoblastomas and non-tumoral liver samples are labeled in bold. Abbreviations: F: Female; M: Male; N/A: not available. ^*^The histology description refers to the frozen tissue sent to DNA extraction. ^#^tumors carrying a *CTNNB1* mutation.

Prior to the differential methylation analyses, several parameters were considered to infer surrogate variables that could impact the comparison of interest. These analyses did not detect any association with the investigated co-variables (age at diagnosis, tumor histology, alpha-fetoprotein levels, treatment protocol, progression and overall survival). The mean and standard deviation of the methylation levels (beta-values ranging from 0 to 1) were very similar within each of the four groups of comparison, Hepatoblastomas set #1 (0.49 +/- 0.37) and set #2 (0.48 +/- 0.37), and control groups of non-tumor differentiated liver set #1 (0.52 +/- 0.36) and set #2 (0.52 +/- 0.36), indicating similar levels of inter- and intra-group heterogeneity, i.e., among the different samples of hepatoblastomas and the different samples of differentiated livers.

**Figure 1:**
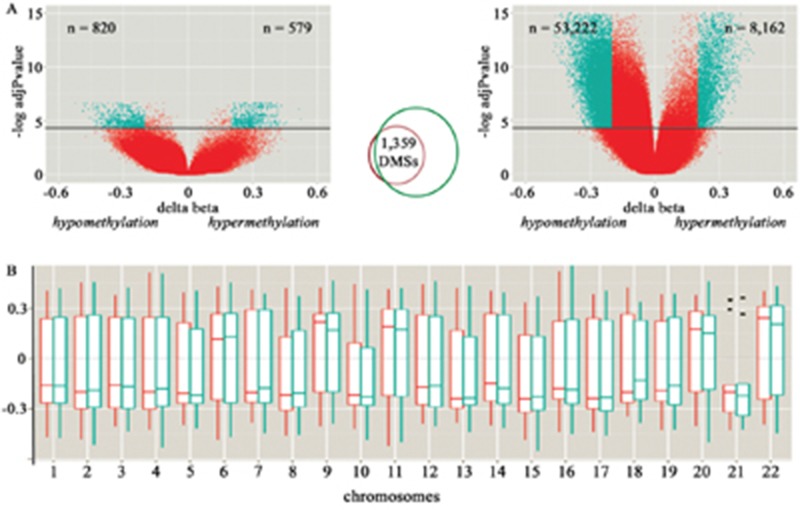
Characterization of the differentially methylated CpG sites (DMSs) detected in hepatoblastomas compared to control liver samples **A.** Volcano plots of the paired (set#1, at left) and non-paired (set #2, at right) differential methylation analysis of hepatoblastomas in comparison to control liver tissues. The X and Y axes display, respectively, the delta-beta value (methylation differences between groups), and the log of the adjusted p-values for each CpG site; above the horizontal black lines, CpG sites considered to be differentially methylated (adjP<0.05). The green dots identify CpG sites displaying >20% methylation difference between tumor and control samples; the number of hypo and hypermethylated sites are indicated by the “n” values displayed, respectively, at left and right top of each Volcano plot. The Venn-diagram in the middle shows the overlapping DMSs that were detected both in set #1 and set #2 of hepatoblastomas. **B.** Distribution of the delta beta mean values (Y axis) of the set of 1,359 common DMSs across the autosomes (rectangular boxes), their corresponding standard deviations (vertical bars) and median values (horizontal bar). Paired (red) and non-paired (green) analyses are shown separately. Outliers are represented by dots.

The comparison between hepatoblastomas and their matched non-tumoral liver tissues from set #1 revealed 1,399 differentially methylated CpG sites (adjP<0.05) – DMSs -, with 820 hypomethylated CpGs (551 of them related to 457 genes), and 579 hypermethylated CpGs (456 of them related to 363 genes) (Figure 1A, left). A validation set of 12 hepatoblastomas (Hepatoblastoma set #2) was used to replicate the analysis, using as a control group the same non-tumoral liver samples plus additional three samples. The set #2 comparison exhibited 61,384 DMSs (adjP<0.05) with a large proportion of them (86.7%; 53,222 CpGs) showing hypomethylation. There was an overlap of 1,359 DMSs (97% of the CpGs detected in the paired differential methylation analysis) between both analyses (Figure 1A; Supplementary Table 1) affecting 765 genes; common DMSs were either hypermethylated or hypomethylated CpGs in both hepatoblastoma sets, with a maximum beta-value variation of 0.21, and an average deviation of 0.009. Unsupervised hierarchical clustering analysis based on the methylation levels of these common 1,359 DMSs discriminated all control livers from 18 out of 19 hepatoblastoma samples (Supplementary Figure 1), except for tumor sample HB17. We did not obtain a clear discrimination of tumor subgroups based on the clinical characteristics of the patients, but given the small size of the cohort, this observation is not conclusive.

The common set of DMSs displayed a non-random distribution regarding different categories of genomic sequences being mostly hypermethylated at gene bodies and CpG islands, and hypomethylated at intergenic regions (p<0.0001; chi-square distribution test, Table 2). Additionally, we inspected the distribution of the DMSs across the autosomes considering the paired and non-paired analyses separately, seeking to uncover a preferential gain or loss of methylation in a specific chromosome (Figure 1B). The autosomes in which the differences of the DMSs were positive (i.e. meaning that the CpG sites were more methylated in hepatoblastomas compared with non-tumoral livers) were chromosomes 6, 9, 11, 20 and 22. Therefore, most of the DMSs were hypomethylated in the majority of the chromosomes, suggesting a widespread and non-randomic pattern of hypomethylation.

**Table 2:** Distribution of the common set of DMSs detected in hepatoblastomas compared to control livers regarding their genomic location

In relation to gene					
	Hypomethylated		Hypermethylated		*p-value*
***1stExon***	19	2%	9	2%	0.4381
***3'UTR***	21	3%	38	7%	0.0003
***5'UTR***	80	10%	48	9%	0.3977
***Gene body***	270	34%	250	45%	<0.0001^*^
***TSS1500***	100	12%	70	13%	1,00
***TSS200***	46	6%	27	5%	0.5412
***Intergenic region***	265	33%	116	21%	0.0001^*^
**In relation to CpG island**					
	**Hypomethylated**		**Hypermethylated**		***p-value***
***Island***	62	8%	81	15%	<0.0001^*^
***N_Shelf***	31	4%	35	06%	0.0537
***N_Shore***	113	14%	71	13%	0.5192
***S_Shelf***	51	6%	36	06%	1,00
***S_Shore***	119	15%	55	10%	0.0065
***Open sea***	425	53%	280	50%	0.3206

Hypomethylated and hypermethylated CpG sites are classified in both gene and CpG island categories and shown in number and percentage. ^*^ Asterisks indicate either gene or CpG island category which we considered statiscally enriched within the group of DMSs (p<0.0001; chi-square distribution test).

DNA methylation is considered a repressive epigenetic mark that characterizes most CpG sites within mammalian genomes. However, CpG dense regions, mainly CpG islands, are generally unmethylated in normal cells [28]. Additionally, DNA methylation values represent a mix of different cell types, thus, small methylation changes may correspond to an epigenetic drift of a subset of cells. Therefore, trying to identify CpG sites with more impact for the disease as a whole, we used an additional threshold of ≥20% methylation difference in hepatoblastomas compared to control livers. We defined differentially methylated regions (DMRs) based on sequences containing ≥3 significant CpG sites, all in the same direction (hypomethylation or hypermethylation) in the same genomic location, with at least one site displaying methylation differences of ≥20%. Thirty-eight genes were found to harbor sequences that met these parameters (Table 3; Supplementary Table 2 contains the details of the CpG sites which are related to DMRs). Among these 38 genes, 14 exhibited hypermethylated CpGs, and 23 exhibited hypomethylation, and one gene, *TSPAN9*, showed both gene body hypermethylation and promoter hypomethylation. One of these genes, *EIF4E*, has already been reported in liver cancer, and other 27 are directly related to liver function.

**Table 3:** Description of the genes associated with 38 differentially methylated regions detected in hepatoblastomas compared to control liver samples

Gene	Methylation change	Gene category (CpG number)	CpG island category (CpG number)	^*^CpG island identification	Description
*ACSL1*	hypermethylation	gene body (3 CpGs) and 5' UTR (1 CpG)	island (2 CpGs) and shores (2 CpGs)	chr4:185724434- 185724647	long-chain fatty-acid-coenzyme A ligase that plays a key role in lipid biosynthesis and fatty acid degradation
*ASCL2*	hypermethylation	TSS1500 (12 CpGs)	island (6 CpGs) and S-shore (6 CpGs)	chr11:2290104- 2292932	transcription factor related to embryonic and induced pluripotent stem cell differentiation, and early embryo development
*CRP*	hypermethylation	TSS200 (1 CpG), TSS1500 (1 CpG), 5'UTR (1 CpG)	open sea (3 CpGs)	N/A	this gene belongs to the pentaxin family, and is involved in several host defense related functions
*DEAF1*	hypermethylation	gene body (3 CpGs)	open sea (3 CpGs)	N/A	regulator of transcription in embryonic development
*ELFN1*	hypermethylation	TSS200 (4 CpGs) and TSS1500 (3 CpGs)	open sea (7 CpGs)	N/A	phosphatase binding and protein phosphatase inhibitor activity
*FAM50B*	hypermethylation	TSS1500 (3 CpGs)	N-shore (3 CpGs)	chr6:3849271-3851048	imprinted gene adjacent to a differentially methylated region (DMR); paternally expressed in many tissues
*HOXA3*	hypermethylation	5' UTR (3 CpGs)	island (1 CpG) and N-shore (2 CpGs)	chr7:27150030- 27150418	homeobox gene whose expression is spatially and temporally regulated during embryonic development
*NUAK1*	hypermethylation	gene body (3 CpGs)	open sea (3 CpGs)	N/A	serine/threonine-protein kinase involved in cell adhesion, regulation of cell ploidy and senescence, and tumor progression
*OXT*	hypermethylation	TSS200 (4 CpGs)	island (4 CpGs)	chr20:3052097- 3053103	encodes a precursor protein that is processed to produce oxytocin and neurophysin I
*PRRT1*	hypermethylation	gene body (10 CpGs) and 3' UTR (4 CpGs)	island (9 CpGs) and N-shore (5 CpGs)	chr6:32118101- 32118544	proline rich transmembrane protein 1
*RXRA*	hypermethylation	gene body (3 CpGs)	island (1 CpG), S-shore (1 CpGs) and open sea (1 CpG)	chr9:137229726- 137229931	nuclear receptor involved in retinoic acid-mediated gene activation
*SNORD46*	hypermethylation	gene body (3 CpGs)	S-shore (3 CpGs)	chr1:45241013- 45241900	small nucleolar RNA
*THRSP*	hypermethylation	TSS200 (2 CpGs), TSS1500 (1 CpG), and first exon (1 CpG)	open sea (4 CpGs)	N/A	gene expressed in liver and adipocytes, with a suggested role in controlling tumor lipid metabolism
*ADAMTS17*	hypomethylation	gene body (3 CpGs)	open sea (3 CpGs)	N/A	disintegrin and metalloproteinase with thrombospondin motifs (ADAMTS family)
*AGL*	hypomethylation	gene body (3 CpGs)	open sea (3 CpGs)	N/A	glycogen debrancher enzyme which is involved in glycogen degradation
*AHRR*	hypomethylation	gene body (3 CpGs)	N-shore (2 CpGs) and open sea (1 CpG)	chr5:370185- 370422	protein of the aryl hydrocarbon receptor (AhR) signaling cascade; involved in regulation of cell growth and differentiation
*ASPSCR1*	hypomethylation	gene body (3 CpGs)	island (2 CpGs) and N-shore (1 CpG)	chr17:79952141-79952494	a tether, which sequesters GLUT4 in the absence of insulin
*BMP4*	hypomethylation	TSS1500 (3 CpGs)	S-shore (3 CpGs)	chr14:54420184-54422958	member of the bone morphogenetic protein (BMP) family of proteins, and of the transforming growth factor-beta (TGF-beta) superfamily; may also be involved in human cancers
*CACNA1H*	hypomethylation	gene body (3 CpGs)	island (2 CpGs) and S-shore (1 CpG)	chr16:1208357- 1208721	protein in the voltage-dependent calcium channel complex
*CACNB4*	hypomethylation	gene body (5 CpGs)	open sea (5 CpGs)		voltage-dependent calcium channel complex protein
*EIF4E*	hypomethylation	5' UTR (4 CpGs)	S-shore (4 CpGs)	chr4:99849305-99850552	component of the eukaryotic translation initiation factor 4F complex; acts as a proto-oncogene
*GHDC*	hypomethylation	TSS200 (3 CpGs)	open sea (3 CpGs)	17:40346266- 40346800	GH3 domain containing
*GRM8*	hypomethylation	gene body (2 CpGs) and TSS200 (1 CpG)	open sea (3 CpGs)		metabotropic glutamate receptor; linked to the inhibition of the cyclic AMP cascade
*IKZF4*	hypomethylation	TSS200 (2 CpGs) and TSS1500 (1 CpG)	open sea (3 CpGs)	N/A	transcription factor, expressed in lymphocytes and implicated in the control of lymphoid development
*KCNMA1*	hypomethylation	gene body (3 CpGs)	open sea (3 CpGs)	N/A	pore-forming subunit of large conductance, voltage and calcium-sensitive potassium channels
*LGALS3BP*	hypomethylation	TSS200 (1 CpG) and TSS1500 (2 CpGs)	open sea (3 CpGs)	17:76975867-76976452	beta-galactoside-binding protein implicated in modulating cell-cell and cell-matrix interactions
*MAP3K8*	hypomethylation	5' UTR (6 CpGs)	S-shore (5 CpGs) and S-shelf (1 CpG)	10:30725901-30727142	oncogene that encodes a member of the serine/threonine protein kinase family
*MRGPRF*	hypomethylation	TSS1500 (3 CpGs)	S-shelf (3 CpGs)	chr11:68778569-68778851	MAS related GPR family member F
*NR2F2*	hypomethylation	TSS200 (5 CpGs), TSS1500 (1 CpGs) and 5' UTR (1 CpG)	S-shelf (7 CpGs)	chr15:96864881-96866787	ligand inducible transcription factor that is involved in the regulation of many different genes
*PFKP*	hypomethylation	gene body (3 CpGs)	S-shore (3 CpGs)	chr10:3148406-3148625	platelet isoform of phosphofructokinase; key regulatory enzyme in glycolysis
*SECTM1*	hypomethylation	gene body (1 CpG) and 3' UTR (3 CpGs)	island (3 CpGs) and N-shelf (1 CpG)	chr17:80278861-80279563	transmembrane and secreted protein thought to be involved in hematopoietic and/or immune system processes
*SLC16A1*	hypomethylation	TSS1500 (4 CpGs)	S-shore (4 CpGs)	chr1:113498366-113499312	proton-linked monocarboxylate transporter across the plasma membrane
*SLC16A5*	hypomethylation	TSS200 (3 CpGs) and TSS1500 (1 CpG)	N-shore (1 CpG) and island (3 CpGs)	chr17:73083866-73084495	protein localized to the cell membrane; acts as a proton-linked transporter of bumetanide
*SP5*	hypomethylation	gene body (4 CpGs) and 3' UTR (1 CpG)	island (4 CpGs) and S-shore (1 CpG)	chr2:171569877-171573904	binds to GC boxes promoter elements; probable regulation of Wnt-mediated beta catenin signaling and target gene transcription
*TINAGL1*	hypomethylation	TSS200 (1 CpG) and 5' UTR (2 CpGs)	open sea (3 CpGs)	N/A	similar to glycoprotein that is recognized by antibodies in some types of immune-related tubulointerstitial nephritis
*TNFRSF19*	hypomethylation	TSS200 (1 CpG) and TSS1500 (2 CpGs)	N-shore (1 CpG) and open sea (2 CpGs)	chr13:24152899-24154140	member of the TNF-receptor superfamily, highly expressed during embryonic development, and capable of inducing apoptosis by a caspase-independent mechanism
*TSPAN9*	hypo/hypermethylation	hypomethylated in 5' UTR (4 CpGs), hypermethylated in gene body (4 CpGs)	hypomethylated in island (4 CpGs), hypermethylated in open sea (4 CpGs)	chr12:3308812-3310270	mediates signal transduction events in the regulation of cell development, activation, growth and motility
*ZBTB38*	hypomethylation	5' UTR (4 CpGs)	open sea (4 CpGs)	N/A	zinc finger transcriptional activator that binds methylated DNA; in mouse, inhibition of this protein has been associated with apoptosis in some cell types

### Differentially methylated genes in hepatoblastomas are involved with liver cell differentiation and cancer

Among the 765 genes associated with the set of common DMSs, there was an enrichment for Metabolic, Cancer, Insulin, MAPK, Hedgehog and Wnt signaling pathways, as well as Regulation of the actin cytoskeleton, Adherens junction, Glycolysis/Gluconeogenesis and Drug Metabolism (Supplementary Table 3). Genes with hypomethylated sites were related to negative regulation of cell differentiation (19 genes), including somatic stem cell maintenance; negative regulation of RNA metabolic processes (32 genes); and negative regulation of DNA-dependent transcription (31 genes). Hypermethylated CpG sites were mapped in genes related to organic acid transport (14 genes), including fatty acid transport; acyl-CoA metabolic processes (8 genes); response to hormone stimulus (28 genes); response to nutrient levels (17 genes); and liver development (8 genes).

Several of the enriched pathways as well as the detected relevant biological processes (liver cell differentiation and stem cell maintenance) might be related to liver development; therefore, we compared the differentially methylated CpG sites detected in hepatoblastomas with the 28,447 CpG sites found to undergo methylation changes during liver development [29]. There were 434 differentially methylated CpG sites associated to 295 genes, this set was found to be significantly enriched during liver development (p<0.0001). Among these 434 CpG sites, 142 (17%) were hypomethylated and 292 (50%) were hypermethylated, with similar patterns of hypomethylation or hypermethylation in both hepatoblastomas and fetal livers compared with adult livers. Functional annotation of the 295 genes revealed enrichment in Metabolic, Cancer, Insulin, MAPK and Wnt signaling pathways, as well as Regulation of the actin cytoskeleton and Adherents junction. Three additional pathways, compared to the whole set of genes, stood out in relevance, namely citrate cycle, tight junction and endocytosis.

**Figure 2:**
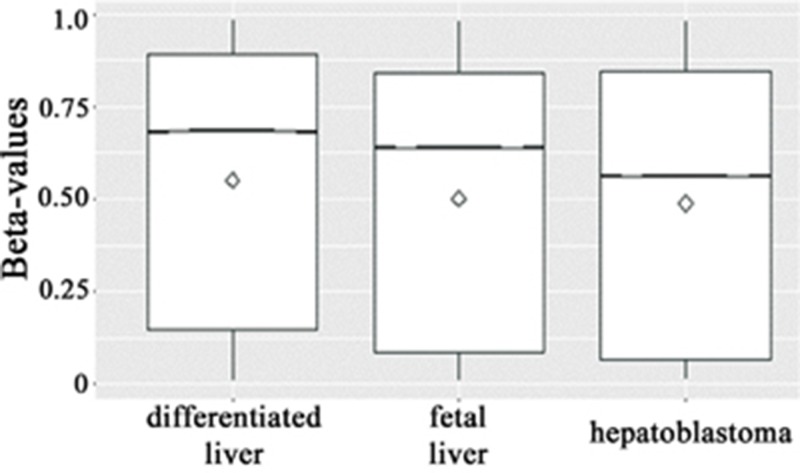
Boxplots of beta-values (25^th^ and 75^th^ percentiles) with notches of all >400,000 CpG sites across the groups (hepatoblastomas, and fetal and differentiated livers) The line and the diamond indicate median and mean methylation values, respectively, and the methylation levels are displayed in the Y axis. Due to the low standard deviation and the large number of beta-values within each group, the notches are not visible. However, the median beta-values of the three groups do not overlap, which is a strong evidence (95% confidence) that the medians are significantly different, indicating that hepatoblastomas are hypomethylated when compared with both fetal and adult differentiated livers.

Therefore, we sought to verify the performance of the global methylation profile in hepatoblastomas in relation to the methylomes of fetal and adult liver samples using this same study [29] as independent groups of liver samples. Beta-values were averaged across samples (hepatoblastomas, fetal or adult livers) and plotted using the notch boxplot method. This method, although not a formal test, infers that if the notches of two boxplots do not overlap, then there is strong evidence (95% confidence) that their medians differ. The results revealed that hepatoblastomas display a significant global hypomethylation pattern when compared with both fetal and adult differentiated livers (Figure 2).

**Figure 3:**
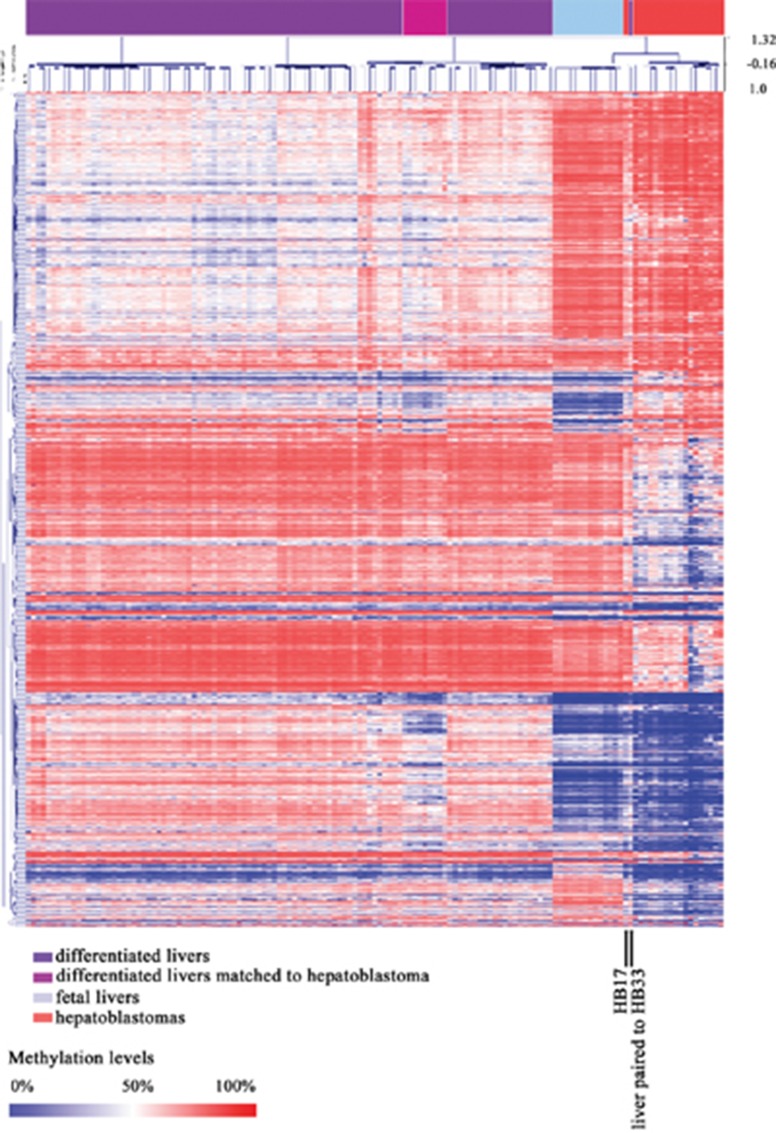
Methylation pattern of combined sample sets from this study (hepatoblastoma and control livers) and Bonder et al, 2014 (fetal and adult liver samples) Heatmap from a non-supervised hierarchical clustering based on the methylation levels of the common set of 1,359 DMSs identified in this study. One non-tumoral liver sample matched to a hepatoblastoma HB33), and one hepatoblastoma sample (HB17) grouped with fetal liver samples (both are indicated by vertical black lines).

Upon unsupervised hierarchical clustering of our set of 1,359 DMSs, two clusters were formed: cluster 1, containing all differentiated liver samples, including those from our study except for one sample, which is a paired non-tumoral sample of a congenital hepatoblastoma (HB33) that grouped with tumors; and cluster 2, which further separated in two branches, one containing all hepatoblastoma samples with exception for HB17, which grouped with all fetal liver samples in the other branch (Figure 3).

Furthermore, we performed an in silico gene expression analysis of the 765 genes associated with the 1,359 differentially methylated CpGs using two studies which contain microarray expression data from hepatoblastomas and non-tumoral liver samples [30, 31]. In the first dataset, there were 717 out of 765 genes in common to both studies, and expression levels of these genes discriminated all non-tumoral liver samples from 94% (47 out of 50) of the hepatoblastoma samples (Supplementary Figure 2A). Using the second dataset, we found that 516 out of 765 genes were common to both studies, i.e., 516 genes harboring differentially methylated sites that were present in the expression microarray; the expression levels exhibited by these common set of genes discriminated all non-tumoral liver samples from 88% (22 out of 25) of the hepatoblastoma samples (Supplementary Figure 2B).

### Profile of methylation in CpGs sites related to retrotransposon sequences (LINE-1) and X-chromosome inactivation

#### LINE-1 sequences are not hypomethylated in hepatoblastomas

**Figure 4:**
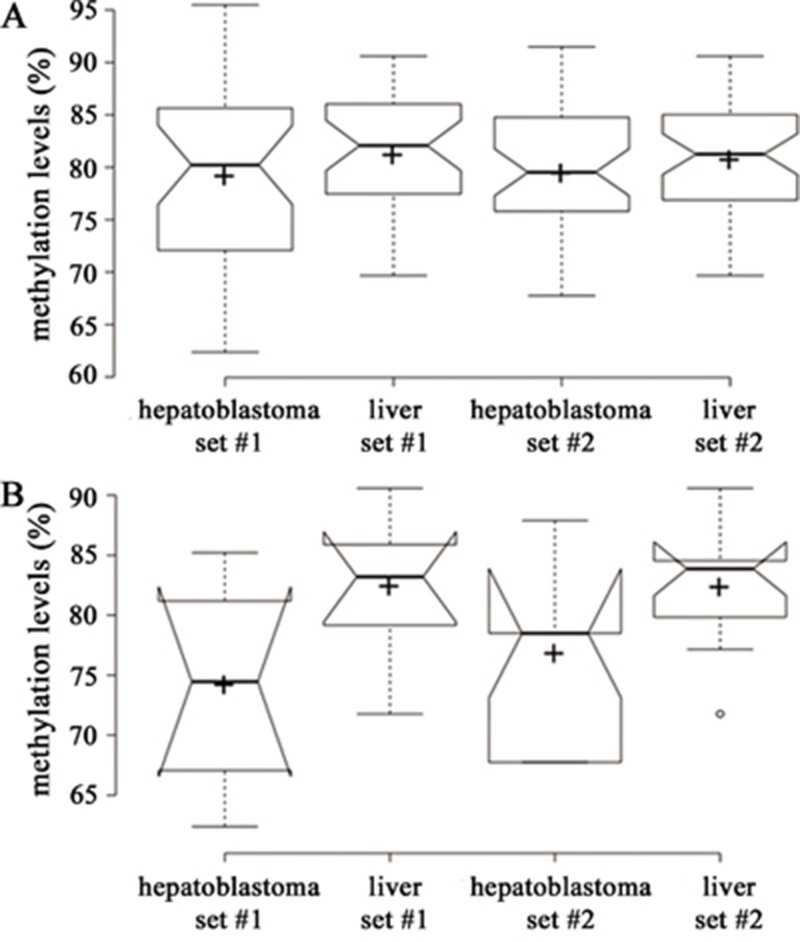
Boxplot of beta-values (25^th^ and 75^th^ percentiles) with notches representing LINE methylation levels The line and the cross indicate median and mean methylation values, respectively, and the methylation levels are displayed in the Y axis. **A.** Methylation levels (%) of all four CpGs located at LINE-1 sequences. **B.** Methylation levels (%) of CpG 1 located at LINE-1 sequences: Hypomethylation of hepatoblastomas set#1 was significant (^*^, <0.05, Student t test) only for the CpG1.

To clarify whether the hypomethylation in hepatoblastomas extends to repetitive sequences, we used pyrosequencing to analyze the methylation status of four CpG sites mapped within LINE-1 sequences. Generally, the LINE-1 methylation levels were very similar across hepatoblastomas and normal differentiated and fetal liver samples. However, focusing on the analysis of individual CpGs, hepatoblastomas from both sets showed a slightly hypomethylated pattern only at the first CpG of the LINE-1 sequence when compared with the differentiated livers (p = 0.03802 for set #1 and p = 0.05174 for set #2; Figure 4). The copy number alteration profile for all samples of hepatoblastoma set #1 has been previously evaluated by array-CGH [32]; tumor samples with and without chromosomal alterations >100 kb were compared with control differentiated livers regarding the methylation levels of LINE-1. Significant differences in LINE-1 methylation levels were found again only for the first CpG site (Supplementary Figure 3), which is slightly hypomethylated in hepatoblastomas with copy number alterations (p = 0.0057; the Kruskall-Wallis test also indicated a difference of p = 0.0166 between the groups at this CpG).

### X-chromosome CpG sites exhibit hypomethylation in male hepatoblastomas

Using all the samples from our study, the methylation levels of CpG sites mapped to the X chromosome were recovered to compare tumor and control liver samples using a sex-matched approach. The methylation profile of the X chromosome was similar in female hepatoblastomas compared with female liver controls, but hypomethylation was detected in male hepatoblastomas (Supplementary Figure 4).

## DISCUSSION

Embryonal tumors are believed to evolve from the disruption of normal embryogenesis [33]. We hypothesized that methylation changes probably contribute to this process, and we analyzed DNA methylation in normal liver and hepatoblastoma samples. Although these are certainly not homogeneous, the methylation data showed minimal variance between samples and groups, indicating that the correction we made before the comparison was efficient. The paired comparison between hepatoblastomas and liver samples revealed few differentially methylated sites exhibiting low-level differences, although the non-paired analysis showed higher differences on top of higher number of sites. This second finding may be related to inherent differences between tumor samples and non-matched controls, reinforcing the contribution of the paired comparison. Notably, the smaller DMS set detected in the paired group is almost completely included in the larger DMS set detected in the non-paired analysis.

Our investigation revealed that hepatoblastomas exhibit a global low-level hypomethylation pattern compared with both normal fetal and differentiated livers. This hypomethylation was generally restricted to non-repetitive DNA, a pattern unusual for most adult solid tumors which exhibit a major hypermethylation at promoters and hypomethylation in repetitive sequences, mainly LINE-1 [34, 35]. However, similar global hypomethylation patterns have recently been reported for hepatocellular carcinoma [32], embryonal tumor medulloblastomas [36] and, lately, in three hepatoblastoma samples [26].

Methylome studies in two other embryonal tumors (Wilms tumor and neuroblastoma) reported a methylation fingerprint in tissue development genes, similar to what we observed in hepatoblastomas. Differentially methylated regions detected in Wilms tumor showed hypomethylation of renal development genes and were enriched for bivalent domains in embryonic stem cells [37]. In neuroblastomas, changes in methylation occurred in sequences related to functional chromatin domains of development and cancer-related genes [38].

Recently, a genome-wide methylation analysis on hepatoblastomas disclosed four novel tumor suppressor genes potentially related to progression in hepatoblastoma, with the increase of methylation levels in more than four genes being associated with poorer prognosis [27].

Functional enrichment analysis of the genes affected by abnormal methylation have highlighted the Wnt signaling pathway, typically altered in hepatoblastomas mainly through a high frequency of *CTNNB1* activating mutations [10, 39]. Here, we found evidence that methylation changes in the genome also interfere with Wnt signaling. Moreover, genes impacted by DMSs were related to pathways that have been associated with cancer, energy and drug metabolism. Glycolysis/gluconeogenesis (GNG) are metabolic pathways reciprocally regulated in the liver that result in the generation of energy. Interestingly, restoration of gluconeogenesis through the inhibition of mTOR appears to improve therapeutic efficacy against hepatocarcinomas [40]. In addition, pathways in cancer, endocytosis and adherens junction were cellular signaling pathways in common previously reported to be deregulated in hepatoblastomas at methylation levels [26]. Regarding the biological processes likely to be impaired by methylation changes, we identified involvement of genes that maintain the interface between the environment and the phenotype, such as those related to the maintenance of nutrient levels and response to hormone stimuli.

To date, it is not clear to what extent the cancer-related epigenetic alterations detected in this study correspond to natural epigenetic variability in human livers; we are aware that fully differentiated liver samples are not an ideal control for methylation studies of embryonal tumors. Therefore, we also compared our data to a recently published study that analyzed methylation changes occurring during liver differentiation [29]. Even with different approaches to determine the DMSs in the two studies, we found an overlap in the frequency and distribution of hypomethylated and hypermethylated CpGs in hepatoblastomas/fetal livers compared with fully differentiated adult livers. The function of such shared CpG sites delineates a stemness profile for hepatoblastoma cells. The respective methylation changes may be related to the postulated impairment of liver cell differentiation leading to the development of hepatic embryonal tumor. It was reported a trend towards hypomethylation in fetal compared to adult liver samples in all CpG sites [29]. Upon clustering, the set of 1,359 DMSs detected in hepatoblastomas clearly showed that tumors and normal fetal livers clustered together into one branch, and all adult liver tissues into another, a finding that supports the hypothesis that hepatoblastomas exhibit a methylation pattern resembling the early stages of liver development. It is likely that hepatoblastoma cells have become fixed in time and space also due to methylation changes, i.e. they have lost their ability to differentiate further into adult liver cells. Adding to this scenario, hepatoblastomas also were reported to display an expression pattern comparable to fetal livers [30].

In our analysis, the signaling pathways related to liver development that may be disturbed by methylation changes in hepatoblastomas were Metabolism, Cancer, Insulin, MAPK and Wnt signaling pathways, as well as Regulation of the actin cytoskeleton and Adherents junction. Recovering publicly available expression data from two independent studies, we showed that the expression levels of the genes associated with the detected differentially methylated CpG sites were able to discriminate all non-tumoral livers from the great majority of the hepatoblastoma samples [30, 31]. This result supports the impact of our methylation findings on tumor gene expression as well as reinforces the role of the detected pathways in the tumorigenic process of hepatoblastoma. Altogether, these data suggest that DNA methylation has a high impact on the expression profile for hepatoblastoma development.

Five of the genes with altered methylation have been previously reported as altered in liver carcinogenesis. *HSD17B13* does not have a known function, but was suggested as a biomarker for hepatocellular carcinomas [41]. *DKK3* is part of the Wnt signaling pathway and is overexpressed in hepatoblastomas, regardless of tumor histology [42]. *MYC* is a transcriptional factor that contributes to liver tumor maintenance [43]. *HAL* is regulated by glucocorticoids and glucagon, and catalyzes the first reaction in histidine metabolism via preferentially the Protein Kinase A (PKA) pathway [44]. *IGF2* is a growth factor frequently overexpressed in hepatoblastomas due to loss of imprinting at 11p15 or paternal uniparental disomy at 11p15 [21].

We found two striking cases in which we believe that exome sequencing would help to further clarify their tumorigenesis processes. One hepatoblastoma (HB17) grouped with the normal liver samples, displaying a similar distribution of methylation levels; one can speculate that in this particular tumor, the occurrence of driver mutations could have triggered the malignant transformation early during liver cells differentiation. Additionally, a non-tumoral liver sample paired with one hepatoblastoma exhibited a methylation pattern more similar to undifferentiated liver cells, grouping with the fetal rather than the differentiated livers, and near tumors in the clustering. This sample was derived from one congenital tumor, suggesting that the methylation changes have been previously established in the non-tumoral liver as a consequence of a highly penetrant germline mutation, that might be related to the epigenetic machinery.

Three genes identified in our hepatoblastoma analyses were also listed in the top 20 differentially methylated genes during liver development: *CRP*, *NNMT* (both hypermethylated) and *C3P1* (hypomethylated). Among them, *NNMT* stood out as an enzyme (nicotinamide N-methyltransferase) that regulates hepatic nutrient metabolism [45]. *NNMT* has also been reported to be overexpressed in a variety of human cancers and to promote epigenetic remodeling in cancer by consuming methyl units from S-adenosyl methionine (SAM), resulting in a metabolic sink [46]. Recently, it has been shown that NNMT and the metabolic state regulate pluripotency in human embryonic stem cells (hESCs) through the consumption of SAM in naive cells, making it unavailable for histone methylation, which represses Wnt and activates the HIF pathway [47]. According to authors, their findings support the hypothesis that the metabolome regulates the epigenetic landscape of the earliest steps in human development. Therefore, we speculate that *NNMT* dysregulation caused by epigenetic changes may be a suitable mechanism linked to the origin of hepatoblastomas, thus deserving further investigation.

Subsequently, we hypothesized that the remaining DMSs detected in hepatoblastomas might be related to cell transformation and tumor progression rather than the maintenance of an undifferentiated cell state. The disease enrichment analyses revealed that ten of these remaining genes have previously been found in liver neoplasms: *THRSP*, *HEPACAM* [48], *MACC1* [49], *TERT* [50], *NUAK1* [51], *NDRG1* [52], *RASSF1* [53], *PRDM2* [54], *ALDOB* [55] and *MT1G* [23], with *MACC1*, *NDRG1* and *RASSF1* already associated with prognosis in liver cancers.

Altogether, we suggest that DNA hypomethylation of intergenic sequences that were primarily detected in hepatoblastomas, as well as the other methylation changes, contributes to the impairment of cell differentiation, which seems to be required for tumorigenesis in embryonal tumors, as reported for meduloblastoma [36], Wilms tumor and neuroblastomas [37, 38]. Alternatively, it may represent an inherent aspect of hepatic tumorigenesis [56]. Both hypotheses are supported by our findings of enrichment for methylation changes occurring in genes relevant for liver differentiation.

Cytosine methylation not only contributes to tissue-specific gene expression but also is associated with the silencing of retrotransposons, genomic imprinting and X-chromosome inactivation in mammals. Therefore, to complement the methylation profile of hepatoblastomas, these aspects were explored. LINE-1 is a retrotransposon repetitive element that constitutes approximately 17% of the human genome. It has been reported to be hypomethylated in many adult tumors, and its methylation level correlates with clinical/pathological features [57, 58]. No differences were found in LINE-1 methylation of hepatoblastomas compared with differentiated livers except for the first LINE-1 CpG, which showed a minor though significant reduction in methylation, similarly to results previously described [17]. Additionally, in our data, low-level hypomethylation of LINE-1 were associated with the occurrence of copy number alterations; when comparing tumors with and without copy number alterations, hypomethylation of the first CpG of LINE-1 was mainly detected in hepatoblastomas harboring chromosomal alterations. However, the absence of significant hypomethylation of repetitive sequences in hepatoblastomas clearly contrasts with most of the solid adult tumors. Therefore, the low-level global hypomethylation reported here is restricted to non-repetitive sequences of the tumors, including intergenic sequences. Such a hypomethylation pattern raises the possibility that intergenic regulatory elements may be affected, as has recently been reported for acute lymphoblastic leukemia [59].

No differences were detected between female hepatoblastomas and control livers regarding the CpG sites located on the X chromosome, suggesting that the X-inactivation, which takes place in the multipotent cell stage during very early embryonic development, was already established when the tumor precursor cells were laid down, and preserved. Conversely, male hepatoblastomas showed a significant loss of methylation at X chromosome CpGs, an observation that needs to be clarified, but that are in accordance with the hypomethylation trend detected in hepatoblastomas in general.

Our results pinpoint that DNA methylation, mostly a low-level hypomethylation, is a key epigenetic mechanism related to hepatoblastoma tumorigenesis, in addition to genetic mutations. It remains to be addressed in future studies whether there is an active process of demethylation working on hepatoblastoma origin, possibly involving TET enzymes and 5hmC, or alternatively, whether a passive pathway of demethylation occurs via inhibition of DNMTs, a possibility previously outlined. Low basal levels of *DNMT1* in hepatoblastoma cell lines confirmed an essential role of *DNMT1* depletion in the enhancement of cancer stem cell properties [60]. The authors have demonstrated that epigenetic reprogramming induced by transient *DNMT1* inhibition influences both malignant properties and the pool of hepatic cancer stem cells.

## CONCLUSIONS

Our results showed that hepatoblastomas exhibit a global low-level hypomethylation pattern in non-repetitive sequences, and an apparent arrest at early stages of liver differentiation. This suggests either that embryonal tumors are driven by different oncogenic mechanisms, as has been observed in other embryonal tumors, or that hypomethylation is a major feature of liver carcinogenesis because it has also been reported in hepatocellular carcinoma. The pathway analysis of the differentially methylated genes revealed an enrichment for metabolism, negative regulation of cell differentiation, and cancer. Moreover, confirming previous genetic findings, the Wnt signaling pathway was highlighted, suggesting a non-stochastic mechanism of CpG methylation in hepatoblastomas. In addition to the methylation landscape, we also provided a list of potential candidate genes for hepatoblastomas.

## MATERIALS AND METHODS

### Human tissue samples

Hepatoblastomas and differentiated non-tumoral liver samples were provided by the Biobank of the A. C. Camargo Cancer Center (ACCCC) and the Pediatric Oncology Institute (GRAACC), which are both cancer hospitals in São Paulo, Brazil. The institutional review board of each institution independently approved the study, and informed consent was obtained from the patients’ legal guardians. All procedures followed the guidelines of the Declaration of Helsinki. All patients received pre-surgical chemotherapy according to SIOPEL (http://www.siopel.org/) or COG (http://www.childrensoncologygroup.org/) protocols. Patients were followed by clinical examination, imaging tests and measurement of alpha-fetoprotein for a minimum period of 18 months.

Sections from tumor tissue blocks stained with hematoxylin and eosin were reviewed by a pathologist. Samples were grouped into a training set (consisting of 8 cases with paired hepatoblastoma and differentiated non-tumoral liver samples; described here as Hepatoblastoma set#1) and a validation set (11 hepatoblastomas and 3 unrelated non-tumoral liver samples - one patient with undisclosed age, the other two patients were 4 and 13 years old; described here as Hepatoblastoma set#2).

### Genomic DNA isolation

Samples were macrodissected for enrichment of neoplastic (at least 70% of homogeneity) or normal tissues, followed by DNA extraction performed at the Macromolecule Bank (A. C. Camargo Cancer Center). Genomic DNA was extracted using phenol:chloroform. NanoDrop (Thermo Fisher Scientific) and Qubit (Thermo Fisher Scientific) equipment were used to assess DNA purity and quantity, and DNA quality was checked by electrophoresis in 0.8% agarose gels.

### DNA methylation analysis

Bisulfite conversion of 500 ng of DNA was performed using the EZ DNA Methylation kit (Zymo Research) according to the manufacturer's recommendations. Bisulfite-converted DNA samples were hybridized in the Human Methylation 450 BeadChip microarrays (HM450K, Illumina), following the Illumina Infinium HD methylation protocol. The HM450K platform measures the DNA methylation level of 485,577 *loci* distributed across the genome at single-nucleotide resolution. Data was submitted to NCBI/GEO and is available as GSE78732.

The Illumina iScan SQ scanner (Illumina) was used to obtain images of the microarrays. The fluorescence signals were interpreted with the GenomeStudio software (v.2011.1) with the methylation module v.1.9.0 (Illumina). Probes were annotated according to the Illumina annotation file using UCSC version hg19 of the human reference genome. The methylation levels for each CpG probe were provided as beta-values ranging from 0 to 1 (0 indicating unmethylated CpGs, and 1 indicating fully methylated CpGs).

*The RnBeads package* [61] was applied to the dataset. A total of 2,001 unreliable probes were identified using Greedycut (p>0.05), and removed. Probes located in SNPs (4,713 probes) as well as those that do not address CpG methylation levels (non-CpG control probes; n=3,140) were also excluded. The background was corrected using the noob method, which is based on a normal-exponential convolution using out-of-band probes [62]. Signal intensities from type I and II probes were normalized using the SWAN method, which adjusts intensities based on a quantile approach [63]. Probes located on the X and Y chromosomes were excluded from downstream analyses, unless otherwise disclosed, resulting in a total of 422,324 normalized β-values for each of the 29 samples (19 hepatoblastomas and 10 control livers). The SVA package [64], that estimates and removes unwanted sources of variation in -omics data, was applied to infer surrogate variables from patient's characteristics (age at diagnosis, tumor histology, alpha-fetoprotein level, treatment protocol, progression and overall survival). Principal Component Analyses (PCA) showed no association between these variables and the groups of comparison regarding methylation levels. However, adjustments for technical effects, such as batch, were performed using default parameters of RnBeads to avoid bias.

### Differential methylation analysis

Beta-values were transformed into M-values before performing comparison between groups, employing an empirical Bayesian framework linear model from limma [65]. CpG sites with adjusted p-values (adjP) <0.05 as defined by Benjamini and Hochberg's method were considered to be differentially methylated. Comparisons were performed in two steps: a paired analysis in 8 cases (Hepatoblastoma set #1), and unpaired analyses comparing an independent set of 11 hepatoblastomas with 3 unpaired non-tumor differentiated liver samples (Hepatoblastoma set #2) plus the same set of 8 non-tumor differentiated liver samples used in the first comparison. The differentially methylated CpG sites (DMSs) that were common to both comparisons (paired and unpaired) were used for downstream analyses. Notch box plots were used to access the distribution of methylation levels of both the DMSs across the autosomal chromosomes, and violin plots were used to evaluate the methylation levels in the X chromosomes of males and females in both cases and controls.

Comparison between wild-type (n=15) and *CTNNB1*-mutated (n=4) tumors were performed using the same parameters. We identified a weak correlation between age at diagnosis (0.48) and alpha-fetoprotein levels (-0.53) with group comparison. Accordingly, adjustment for the M-values of each sample were performed before differential methylation analyses at CpG site levels.

Functional enrichment analyses (Biological Processes from Gene Ontology and cellular signaling pathways from KEGG) were performed using WebGestalt [62] with the whole genome as the background. Features with adjP<0.05 (hypergeometric test with Benjamini–Hochberg adjustment) comprising at least five genes were considered significant.

### Hepatoblastomas and liver development

Next, we compared our data to CpGs reported to exhibit methylation changes during liver development [29]. In the mentioned study, HM450K arrays were used to identify methylation changes between 14 fetal and 96 adult livers, revealing 28,447 differentially methylated CpG sites. These data were compared with the detected DMSs from our study, taking into consideration whether the sites were hypomethylated and hypermethylated. In addition, using Modular Single-set Enrichment Test (MSET), we assessed the enrichment of our DMSs to the differentially methylated CpGs between fetal and adult liver samples [29], generating an *in silico* p-value based on 10,000 random datasets [66].

Furthermore, to obtain a methylation profile of fetal and adult livers, the IDAT files from GEO (GSE61279) were downloaded, preprocessed and normalized using the same analytical procedure applied to our study. There were 420,432 common B-values for both datasets, ours and the afore mentioned liver development study. Average B-values from fetal and adult livers were compared with the average B-values of hepatoblastomas sets #1 and #2.

Pearson correlation with complete linkage was used for non-supervised hierarchical clustering of hepatoblastomas and differentiated and fetal liver samples; reliability was assessed by bootstrapping using multiExperiment Viewer (MeV) software [67].

### Differential gene expression analysis

The 765 genes associated with the 1,359 differentially methylated CpGs detected in our study were searched in two different datasets of expression microarrays, one of them downloaded from ArrayExpress (http://www.ebi.ac.uk/arrayexpress/experiments/E-MEXP-1851/), which contains data of 25 hepatoblastomas and four non-tumoral liver samples [30], and the second set recovered from GEO (http://www.ncbi.nlm.nih.gov/geo/; GSE75271), corresponding to expression data from 50 hepatoblastomas and five non-tumoral liver samples [31]. Expression levels representing the set of genes common to our study were used for a non-supervised hierarchical clustering of hepatoblastomas and non-tumoral liver samples using Pearson correlation with average linkage.

## SUPPLEMENTARY MATERIALS FIGURES AND TABLES









## References

[1] Reik W (2007). Stability and flexibility of epigenetic gene regulation in mammalian development. Nature.

[2] Barker DJ (2007). The origins of the developmental origins theory. J Intern Med.

[3] Hanson MA, Gluckman PD (2008). Developmental origins of health and disease: new insights. Basic Clin Pharmacol Toxicol.

[4] Armstrong DA, Lesseur C, Conradt E, Lester BM, Marsit CJ (2014). Global and gene-specific DNA methylation across multiple tissues in early infancy: implications for children's health research. FASEB J.

[5] Vogelstein B, Papadopoulos N, Velculescu VE, Zhou S, Diaz LA, Kinzler KW (2013). Cancer genome landscapes. Science.

[6] Zhang J, Walsh MF, Wu G, Edmonson MN, Gruber TA, Easton J, Hedges D, Ma X, Zhou X, Yergeau DA, Wilkinson MR, Vadodaria B, Chen X (2015). Germline Mutations in Predisposition Genes in Pediatric Cancer. N Engl J Med.

[7] Maris JM, Denny CT (2002). Focus on embryonal malignancies. Cancer Cell.

[8] Howlader N, Noone AM, Krapcho M, Neyman N, Aminou R, Waldron W, Altekruse SF, Kosary CL, Ruhl J, Tatalovich Z, Cho H, Mariotto A, Eisner MP SEER Cancer Statistics Review, 1975-2008.

[9] de Camargo B, de Oliveira Ferreira JM, de Souza Reis R, Ferman S, de Oliveira Santos M, Pombo-de-Oliveira MS (2011). Socioeconomic status and the incidence of non-central nervous system childhood embryonic tumours in Brazil. BMC Cancer.

[10] Eichenmüller M, Trippel F, Kreuder M, Beck A, Schwarzmayr T, Häberle B, Cairo S, Leuschner I, von Schweinitz D, Strom TM, Kappler R (2014). The genomic landscape of hepatoblastoma and their progenies with HCC-like features. J Hepatol.

[11] Udatsu Y, Kusafuka T, Kuroda S, Miao J, Okada A (2001). High frequency of beta-catenin mutations in hepatoblastoma. Pediatr Surg Int.

[12] Mokkapati S, Niopek K, Huang L, Cunniff KJ, Ruteshouser EC, deCaestecker M, Finegold MJ, Huff V (2014). β-catenin activation in a novel liver progenitor cell type is sufficient to cause hepatocellular carcinoma and hepatoblastoma. Cancer Res.

[13] Lister R, Pelizzola M, Dowen RH, Hawkins RD, Hon G, Tonti-Filippini J, Nery JR, Lee L, Ye Z, Ngo QM, Edsall L, Antosiewicz-Bourget J, Stewart R (2009). Human DNA methylomes at base resolution show widespread epigenomic differences. Nature.

[14] Robertson KD (2005). DNA methylation and human disease. Nat Rev Genet.

[15] Baylin SB, Jones PA (2011). A decade of exploring the cancer epigenome - biological and translational implications. Nat Rev Cancer.

[16] Chatterjee R, Vinson C (2012). CpG methylation recruits sequence specific transcription factors essential for tissue specific gene expression. Biochim Biophys Acta.

[17] Rumbajan JM, Maeda T, Souzaki R, Mitsui K, Higashimoto K, Nakabayashi K, Yatsuki H, Nishioka K, Harada R, Aoki S, Kohashi K, Oda Y, Hata K (2013). Comprehensive analyses of imprinted differentially methylated regions reveal epigenetic and genetic characteristics in hepatoblastoma. BMC Cancer.

[18] Honda S, Haruta M, Sugawara W, Sasaki F, Ohira M, Matsunaga T, Yamaoka H, Horie H, Ohnuma N, Nakagawara A, Hiyama E, Todo S, Kaneko Y (2008). The methylation status of RASSF1A promoter predicts responsiveness to chemotherapy and eventual cure in hepatoblastoma patients. Int J Cancer.

[19] Honda S, Miyagi H, Suzuki H, Minato M, Haruta M, Kaneko Y, Hatanaka KC, Hiyama E, Kamijo T, Okada T, Taketomi A (2013). RASSF1A methylation indicates a poor prognosis in hepatoblastoma patients. Pediatr Surg Int.

[20] Sugawara W, Haruta M, Sasaki F, Watanabe N, Tsunematsu Y, Kikuta A, Kaneko Y (2007). Promoter hypermethylation of the RASSF1A gene predicts the poor outcome of patients with hepatoblastoma. Pediatr Blood Cancer.

[21] Honda S, Arai Y, Haruta M, Sasaki F, Ohira M, Yamaoka H, Horie H, Nakagawara A, Hiyama E, Todo S, Kaneko Y (2008). Loss of imprinting of IGF2 correlates with hypermethylation of the H19 differentially methylated region in hepatoblastoma. Br J Cancer.

[22] Nagai H, Naka T, Terada Y, Komazaki T, Yabe A, Jin E, Kawanami O, Kishimoto T, Konishi N, Nakamura M, Kobayashi Y, Emi M (2003). Hypermethylation associated with inactivation of the SOCS-1 gene, a JAK/STAT inhibitor, in human hepatoblastomas. J Hum Genet.

[23] Sakamoto LH, de Camargo B, Cajaiba M, Soares FA, Vettore AL (2010). MT1G hypermethylation: a potential prognostic marker for hepatoblastoma. Pediatr Res.

[24] Shim YH, Park HJ, Choi MS, Kim JS, Kim H, Kim JJ, Jang JJ, Yu E (2003). Hypermethylation of the p16 gene and lack of p16 expression in hepatoblastoma. Mod Pathol.

[25] Regel I, Eichenmüller M, Joppien S, Liebl J, Häberle B, Müller-Höcker J, Vollmar A, von Schweinitz D, Kappler R (2012). IGFBP3 impedes aggressive growth of pediatric liver cancer and is epigenetically silenced in vascular invasive and metastatic tumors. Mol Cancer.

[26] Cui X, Liu B, Zheng S, Dong K, Dong R (2016). Genome-wide analysis of DNA methylation in hepatoblastoma tissues. Oncol Lett.

[27] Honda S, Minato M, Suzuki H, Fujiyoshi M, Miyagi H, Haruta M, Kaneko Y, Hatanaka KC, Hiyama E, Kamijo T, Okada T, Taketomi A (2016). Clinical prognostic value of DNA methylation in hepatoblastoma: four novel tumor suppressor candidates. Cancer Sci.

[28] Deaton AM, Bird A (2011). CpG islands and the regulation of transcription. Genes Dev.

[29] Bonder MJ, Kasela S, Kals M, Tamm R, Lokk K, Barragan I, Buurman WA, Deelen P, Greve JW, Ivanov M, Rensen SS, van Vliet-Ostaptchouk JV, Wolfs MG (2014). Genetic and epigenetic regulation of gene expression in fetal and adult human livers. BMC Genomics.

[30] Cairo S, Armengol C, De Reyniès A, Wei Y, Thomas E, Renard CA, Goga A, Balakrishnan A, Semeraro M, Gresh L, Pontoglio M, Strick-Marchand H, Levillayer F (2008). Hepatic stem-like phenotype and interplay of Wnt/beta-catenin and Myc signaling in aggressive childhood liver cancer. Cancer Cell.

[31] Sumazin P, Chen Y, Treviño LR, Sarabia SF, Hampton OA, Patel K, Mistretta TA, Zorman B, Thompson P, Heczey A, Comerford S, Wheeler DA, Chintagumpala M (2017). Genomic analysis of hepatoblastoma identifies distinct molecular and prognostic subgroups. Hepatology.

[32] Rodrigues TC, Fidalgo F, da Costa CM, Ferreira EN, da Cunha IW, Carraro DM, Krepischi AC, Rosenberg C (2014). Upregulated genes at 2q24 gains as candidate oncogenes in hepatoblastomas. Future Oncol.

[33] Rushton J, López-Terrada D (2010). Molecular and genetic basis of childhood cancer. Cancer Biomark.

[34] Heyn H, Esteller M (2012). DNA methylation profiling in the clinic: applications and challenges. Nat Rev Genet.

[35] Miousse IR, Koturbash I (2015). The Fine LINE: Methylation Drawing the Cancer Landscape. BioMed Res Int.

[36] Hovestadt V, Remke M, Kool M, Pietsch T, Northcott PA, Fischer R, Cavalli FM, Ramaswamy V, Zapatka M, Reifenberger G, Rutkowski S, Schick M, Bewerunge-Hudler M (2013). Robust molecular subgrouping and copy-number profiling of medulloblastoma from small amounts of archival tumour material using high-density DNA methylation arrays. Acta Neuropathol.

[37] Charlton J, Williams RD, Sebire NJ, Popov S, Vujanic G, Chagtai T, Alcaide-German M, Morris T, Butcher LM, Guilhamon P, Beck S, Pritchard-Jones K (2015). Comparative methylome analysis identifies new tumour subtypes and biomarkers for transformation of nephrogenic rests into Wilms tumour. Genome Med.

[38] Gómez S, Castellano G, Mayol G, Suñol M, Queiros A, Bibikova M, Nazor KL, Loring JF, Lemos I, Rodríguez E, de Torres C, Mora J, Martín-Subero JI, Lavarino C (2015). DNA methylation fingerprint of neuroblastoma reveals new biological and clinical insights. Epigenomics.

[39] Kosaki R, Takenouchi T, Takeda N, Kagami M, Nakabayashi K, Hata K, Kosaki K (2014). Somatic CTNNB1 mutation in hepatoblastoma from a patient with Simpson-Golabi-Behmel syndrome and germline GPC3 mutation. Am J Med Genet A.

[40] Khan MW, Biswas D, Ghosh M, Mandloi S, Chakrabarti S, Chakrabarti P (2015). mTORC2 controls cancer cell survival by modulating gluconeogenesis. Cell Death Dis.

[41] Xing X, Huang Y, Wang S, Chi M, Zeng Y, Chen L, Li L, Zeng J, Lin M, Han X, Liu X, Liu J (2015). Comparative analysis of primary hepatocellular carcinoma with single and multiple lesions by iTRAQ-based quantitative proteomics. J Proteomics.

[42] Pei Y, Kano J, Iijima T, Morishita Y, Inadome Y, Noguchi M (2009). Overexpression of Dickkopf 3 in hepatoblastomas and hepatocellular carcinomas. Virchows Arch.

[43] Kress TR, Pellanda P, Pellegrinet L, Bianchi V, Nicoli P, Doni M, Recordati C, Bianchi S, Rotta L, Capra T, Ravà M, Verrecchia A, Radaelli E (2016). Identification of MYC-Dependent Transcriptional Programs in Oncogene-Addicted Liver Tumors. Cancer Res.

[44] Alemán G, Ortíz V, Langley E, Tovar AR, Torres N (2005). Regulation by glucagon of the rat histidase gene promoter in cultured rat hepatocytes and human hepatoblastoma cells. Am J Physiol Endocrinol Metab.

[45] Hong S, Moreno-Navarrete JM, Wei X, Kikukawa Y, Tzameli I, Prasad D, Lee Y, Asara JM, Fernandez-Real JM, Maratos-Flier E, Pissios P (2015). Nicotinamide N-methyltransferase regulates hepatic nutrient metabolism through Sirt1 protein stabilization. Nat Med.

[46] Ferreri AJ, Illerhaus G, Zucca E, Cavalli F, International Extranodal Lymphoma Study Group (2010). Flows and flaws in primary central nervous system lymphoma. Nat Rev Clin Oncol.

[47] Sperber H, Mathieu J, Wang Y, Ferreccio A, Hesson J, Xu Z, Fischer KA, Devi A, Detraux D, Gu H, Battle SL, Showalter M, Valensisi C (2015). The metabolome regulates the epigenetic landscape during naive-to-primed human embryonic stem cell transition. Nat Cell Biol.

[48] Chung Moh M, Hoon Lee L, Shen S (2005). Cloning and characterization of hepaCAM, a novel Ig-like cell adhesion molecule suppressed in human hepatocellular carcinoma. J Hepatol.

[49] Xie C, Wu J, Yun J, Lai J, Yuan Y, Gao Z, Li M, Li J, Song L (2013). MACC1 as a prognostic biomarker for early-stage and AFP-normal hepatocellular carcinoma. PLoS One.

[50] Kim H, Yoo JE, Cho JY, Oh BK, Yoon YS, Han HS, Lee HS, Jang JJ, Jeong SH, Kim JW, Park YN (2013). Telomere length, TERT and shelterin complex proteins in hepatocellular carcinomas expressing “stemness”-related markers. J Hepatol.

[51] Cui J, Yu Y, Lu GF, Liu C, Liu X, Xu YX, Zheng PY (2013). Overexpression of ARK5 is associated with poor prognosis in hepatocellular carcinoma. Tumour Biol.

[52] Cheng J, Xie HY, Xu X, Wu J, Wei X, Su R, Zhang W, Lv Z, Zheng S, Zhou L (2011). NDRG1 as a biomarker for metastasis, recurrence and of poor prognosis in hepatocellular carcinoma. Cancer Lett.

[53] Zhang C, Li J, Huang T, Duan S, Dai D, Jiang D, Sui X, Li D, Chen Y, Ding F, Huang C, Chen G, Wang K (2016). Meta-analysis of DNA methylation biomarkers in hepatocellular carcinoma. Oncotarget.

[54] Piao GH, Piao WH, He Y, Zhang HH, Wang GQ, Piao Z (2008). Hyper-methylation of RIZ1 tumor suppressor gene is involved in the early tumorigenesis of hepatocellular carcinoma. Histol Histopathol.

[55] Wu J, Dan C, Zhao HB, Xiao CX, Liu YP, Si LJ, Ren JL, Guleng B (2014). ALDOB acts as a novel HBsAg-binding protein and its coexistence inhibits cisplatin-induced HepG2 cell apoptosis. Crit Rev Eukaryot Gene Expr.

[56] Shen J, Wang S, Zhang YJ, Wu HC, Kibriya MG, Jasmine F, Ahsan H, Wu DP, Siegel AB, Remotti H, Santella RM (2013). Exploring genome-wide DNA methylation profiles altered in hepatocellular carcinoma using Infinium HumanMethylation 450 BeadChips. Epigenetics.

[57] Baba Y, Huttenhower C, Nosho K, Tanaka N, Shima K, Hazra A, Schernhammer ES, Hunter DJ, Giovannucci EL, Fuchs CS, Ogino S (2010). Epigenomic diversity of colorectal cancer indicated by LINE-1 methylation in a database of 869 tumors. Mol Cancer.

[58] Beck CR, Collier P, Macfarlane C, Malig M, Kidd JM, Eichler EE, Badge RM, Moran JV (2010). LINE-1 retrotransposition activity in human genomes. Cell.

[59] Almamun M, Levinson BT, van Swaay AC, Johnson NT, McKay SD, Arthur GL, Davis JW, Taylor KH (2015). Integrated methylome and transcriptome analysis reveals novel regulatory elements in pediatric acute lymphoblastic leukemia. Epigenetics.

[60] Clark AT (2015). DNA methylation remodeling in vitro and in vivo. Curr Opin Genet Dev.

[61] Assenov Y, Müller F, Lutsik P, Walter J, Lengauer T, Bock C (2014). Comprehensive analysis of DNA methylation data with RnBeads. Nat Methods.

[62] Triche TJ, Weisenberger DJ, Van Den Berg D, Laird PW, Siegmund KD (2013). Low-level processing of Illumina Infinium DNA Methylation BeadArrays. Nucleic Acids Res.

[63] Maksimovic J, Gordon L, Oshlack A (2012). SWAN: subset-quantile within array normalization for illumina infinium HumanMethylation450 BeadChips. Genome Biol.

[64] Leek JT, Johnson WE, Parker HS, Jaffe AE, Storey JD (2012). The sva package for removing batch effects and other unwanted variation in high-throughput experiments. Bioinformatics.

[65] Ritchie ME, Phipson B, Wu D, Hu Y, Law CW, Shi W, Smyth GK (2015). limma powers differential expression analyses for RNA-sequencing and microarray studies. Nucleic Acids Res.

[66] Eisinger BE, Saul MC, Driessen TM, Gammie SC (2013). Development of a versatile enrichment analysis tool reveals associations between the maternal brain and mental health disorders, including autism. BMC Neurosci.

[67] Saeed AI, Sharov V, White J, Li J, Liang W, Bhagabati N, Braisted J, Klapa M, Currier T, Thiagarajan M, Sturn A, Snuffin M, Rezantsev A (2003). TM4: a free, open-source system for microarray data management and analysis. Biotechniques.

