# Supplementary material for: DNA methylation landscape of hepatoblastomas reveals arrest at early stages of liver differentiation and cancer-related alterations
Source: Oncotarget. 2016 Dec 25;8(58):97871–89. doi: 10.18632/oncotarget.14208 (PMC5716698; doi:10.18632/oncotarget.14208)
Supplement: Supplementary file 1 [file oncotarget-08-97871-s001.pdf]

DNA methylation landscape of hepatoblastomas reveals arrest at early stages of liver differentiation and cancer-related alterations

SUPPLEMENTARY MATERIALS

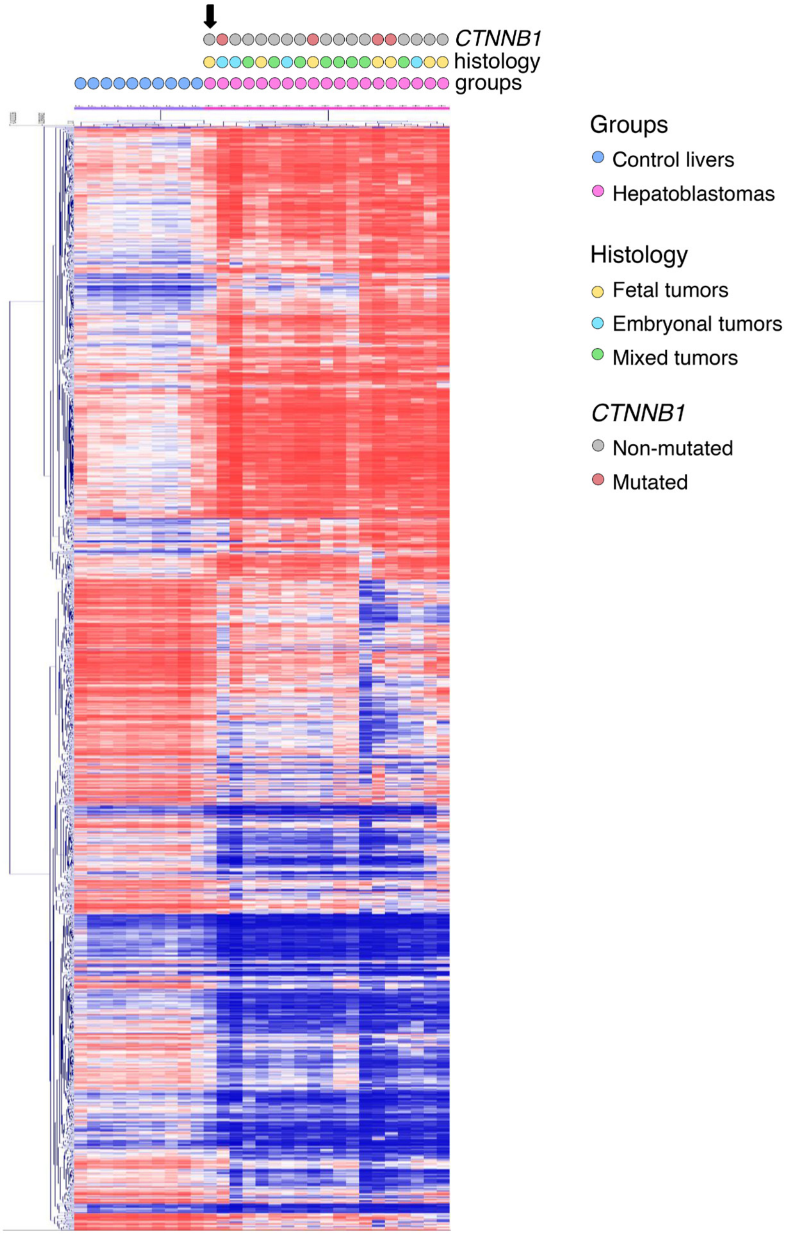

Supplementary Figure 1: Non-supervised hierarchical clustering of 19 hepatoblastomas samples and 10 control liver samples based on the methylation levels of the common set of 1,359 differentially methylated CpG sites (DMSs). Control livers were grouped separately from 18 of the 19 hepatoblastoma samples (with exception of the tumor sample HB17, which is indicated by a vertical black arrow). The methylation pattern did not distinguish tumor samples according to their histological classification. Legend of colors at right indicates selected characteristics of the hepatoblastomas.

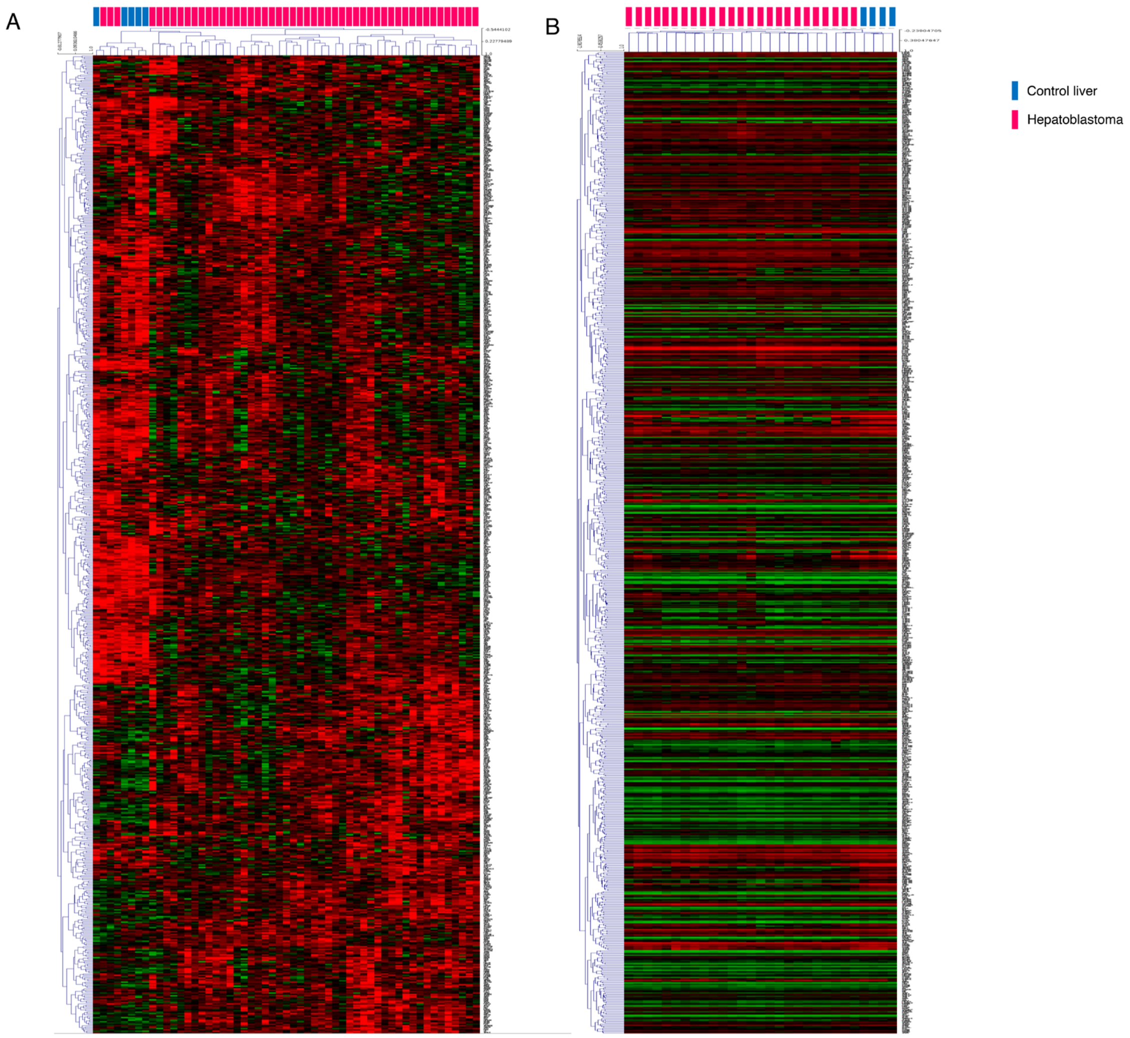

Supplementary Figure 2: Non-supervised hierarchical clustering based on the expression levels of genes harboring differentially methylated CpG sites. A. Cluster derived from microarray expression data of 50 hepatoblastomas and 5 liver samples, containing 717 genes in common to our results [31]. **B.** Cluster derived from microarray expression data of 25 hepatoblastomas and 4 liver samples, containing 516 genes in common to our results [30]. Legend of colors at right indicates tumor and non-tumoral liver samples.

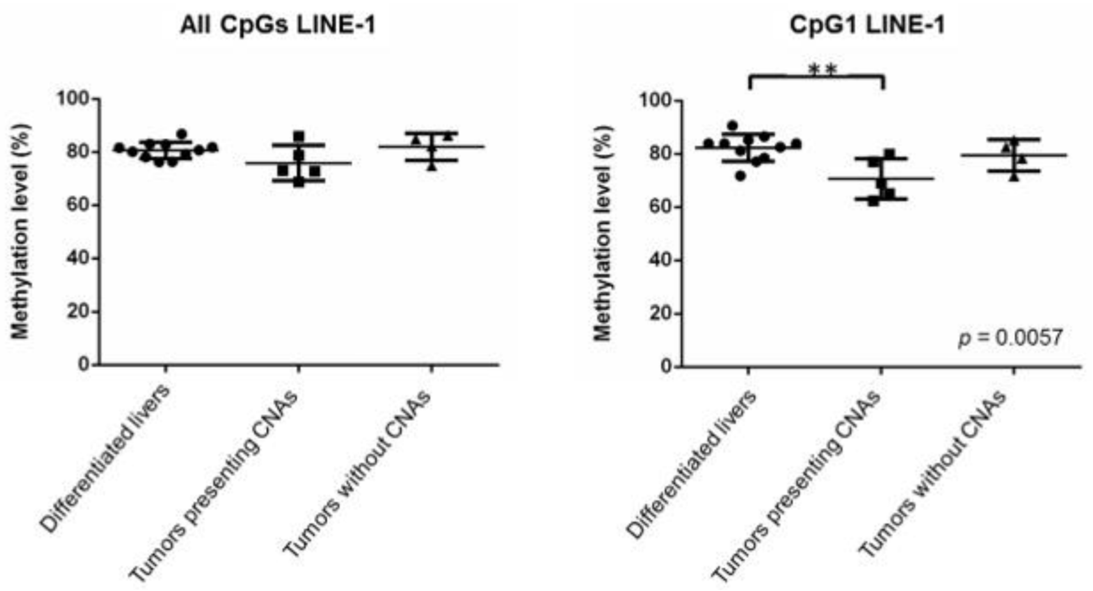

Supplementary Figure 3. LINE-1 methylation levels of hepatoblastomas (set#1) with and without chromosomal alterations >100 kb. Significant differences in LINE-1 methylation levels were found at the first CpG site, which is slightly hypomethylated in hepatoblastomas with copy number alterations ($p = 0.0057$).

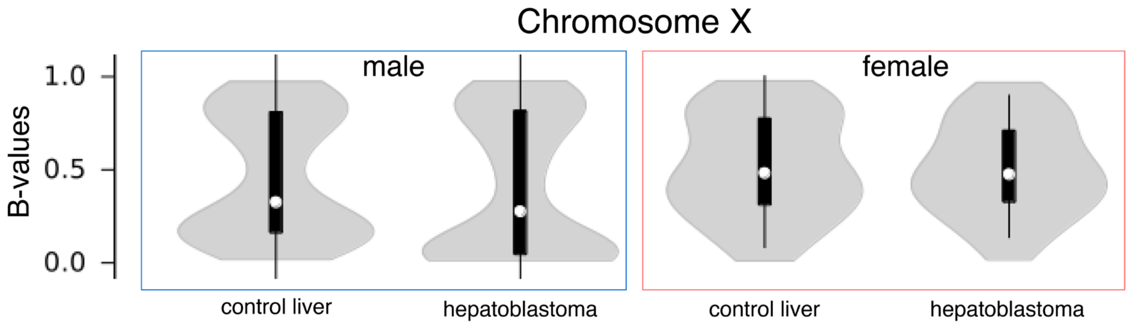

Supplementary Figure 4: Methylation levels of CpG sites mapped on the X-chromosome in hepatoblastomas and control liver samples grouped by gender. The violin plots show the distribution of the methylation levels of CpG sites. Thick vertical bars represent the B-values density and their limits indicate the 25th and 75th percentiles; white circles represent the median and thin bars extend to extreme values.

Supplementary Table 1: The common set of 1,359 CpG differentially methylated sites between hepatoblastomas and control liver samples in paired and non-paired sets of samples. Cg_id labeled in red or blue are hypomethylated and hypermethylated sites, respectively

See Supplementary File 1

Supplementary Table 2: Description of the CpG sites contained in the 38 differentially methylated regions (DMRs) defined as sequences containing ≥3 significant CpG sites with at least one showing methylation differences ≥20%, all with methylation differences in the same direction. Cg_ids labeled in red or blue are hypomethylated and hypermethylated sites, respectively

See Supplementary File 2

Supplementary Table 3: Functional enrichment analyses: biological processes from Gene Ontology and cellular signaling pathways from KEGG (shown in different spreadsheets) performed using WebGestalt with the whole genome as background. The enriched pathways as well as relevant biological processes related to the 765 genes associated with the DMSs detected in hepatoblastomas are shown

See Supplementary File 3
